# Innovative Education to Career Pipelines in Long-Term Care: The Scholars With A.G. Rhodes Program

**DOI:** 10.1093/geroni/igaf122.766

**Published:** 2025-12-31

**Authors:** Wendy Simonds, Jennifer Morgan, Antonius Skipper

**Affiliations:** Georgia State University, Atlanta, Georgia, United States; Georgia State University, Atlanta, Georgia, United States; Georgia State University, Atlanta, Georgia, United States

## Abstract

Gerontology programs often seek to enter into education-practice partnerships to support students to gain hands-on knowledge and experience that prepares them for careers serving older adults. This collaboration, known as “Scholars With A.G. Rhodes at GSU” (SWAGR at GSU), offers master’s students the opportunity to gain valuable hands-on experience in long-term care settings while completing their graduate training in Gerontology. By combining education with real-world practice, the program aims to strengthen the elder care workforce, enrich the students’ professional development, and elaborate a research-practice partnership. A.G. Rhodes has committed to helping students remain working in its centers after graduation. The SWAGR program extends the usual partnerships that foreground service learning or internships while seeking to create educational/career pipelines for international students with undergraduate nursing degrees. The program not only helps fill the growing need for skilled elder care professionals but also provides a much-needed pathway to establish nursing careers in nursing homes. Our presentation will describe the pilot program, in which nine students participated during the first year. Most SWAGRs served as “non-clinical interns” and assisted with resident engagement programs; two who had already passed their NCLEX exams did more traditional clinical internships. We will discuss the challenges and accomplishments the students experienced on site at three A.G. Rhodes centers. We will also discuss our plans to develop a program of research embedded in A.G. Rhodes to support model development, translation of research to practice and engaged scholarship.

